# MMP1, IL-1β, sTNFR-1, and IL-6 are prognostic factors for patients with unresectable or metastatic renal cell carcinoma treated with immune checkpoint inhibitors

**DOI:** 10.1007/s10147-024-02477-4

**Published:** 2024-04-06

**Authors:** Hirotaka Nagasaka, Takeshi Kishida, Taku Kouro, Yuka Igarashi, Shinichi Takebe, Shotaro Yamamoto, Takuya Kondo, Mitsuyuki Koizumi, Hideyuki Terao, Takahisa Suzuki, Noboru Nakaigawa, Hidetomo Himuro, Feifei Wei, Tetsuro Sasada

**Affiliations:** 1 Department of Urology, Kanagawa Cancer Center, Yokohama, Kanagawa 241-8515 Japan; 2 Division of Cancer Immunotherapy, Kanagawa Cancer Center Research Institute, Yokohama, Kanagawa 241-8515 Japan; 3 Cancer Vaccine and Immunotherapy Center, Kanagawa Cancer Center, Kanagawa, Japan

**Keywords:** Renal cell carcinoma, Immune checkpoint inhibitor (ICI), Prognostic factor, Immune-related adverse events (irAE), MMP1, IL-6

## Abstract

**Background:**

Few studies have reported reliable prognostic factors for immune checkpoint inhibitors (ICIs) in renal cell carcinoma (RCC). Therefore, we investigated prognostic factors in patients treated with ICIs for unresectable or metastatic RCC.

**Methods:**

We included 43 patients who received ICI treatment for RCC between January 2018 and October 2021. Blood samples were drawn before treatment, and 73 soluble factors in the plasma were analyzed using a bead-based multiplex assay. We examined factors associated with progression-free survival (PFS), overall survival (OS), and immune-related adverse events (irAE) using the Chi-squared test, Kaplan–Meier method, and the COX proportional hazards model.

**Results:**

Patients exhibited a median PFS and OS of 212 and 783 days, respectively. Significant differences in both PFS and OS were observed for MMP1 (PFS, p < 0.001; OS, p = 0.003), IL-1β (PFS, p = 0.021; OS, p = 0.008), sTNFR-1 (PFS, p = 0.017; OS, p = 0.005), and IL-6 (PFS, p = 0.004; OS, p < 0.001). Multivariate analysis revealed significant differences in PFS for MMP1 (hazard ratio [HR] 5.305, 95% confidence interval [CI], 1.648–17.082; p = 0.005) and OS for IL-6 (HR 23.876, 95% CI, 3.426–166.386; p = 0.001). Moreover, 26 patients experienced irAE, leading to ICI discontinuation or withdrawal. MMP1 was significantly associated with irAE (p = 0.039).

**Conclusion:**

MMP1 may be associated with severe irAE, and MMP1, IL-1β, sTNFR-1, and IL-6 could serve as prognostic factors in unresectable or metastatic RCC treated with ICIs. MMP1 and IL-6 were independent predictors of PFS and OS, respectively. Thus, inhibiting these soluble factors may be promising for enhancing antitumor responses in patients with RCC treated with ICIs.

## Introduction

An estimated 403,000 people were diagnosed with renal cell carcinoma (RCC) in 2018, accounting for 2.2% of all cancer diagnoses. Treatment of metastatic RCC is based on drug therapy when metastases are numerous or when the primary tumor is unresectable. The 5-year survival rate for metastatic RCC is as low as 12% [1]. Drug therapy often includes the use of molecularly targeted drugs and immune checkpoint inhibitors (ICIs). Understanding the optimal treatment for individual patients at specific stages is crucial, highlighting the importance of identifying and considering prognostic factors. Notably, RCC prognostic score models, such as the International Metastatic RCC Database Consortium (IMDC) risk classification and The Memorial Sloan Kettering Cancer Center (MSKCC) prognostic models, are well established [2, 3].

In RCC, there is an association between inflammation and cancer cell growth and invasion. For this reason, inflammation-related factors, such as peripheral blood cells and C-reactive protein (CRP), have been reported as prognostic factors in previous studies [4–6]. Most of these predictors are prognostic factors for patients with unresectable or metastatic RCC treated with molecularly targeted drugs or interferons.

Owing to the emergence and increased availability of ICIs for the treatment of RCC, the importance of prognostic factors has also increased given the increased complexity of treatment decisions. However, there is a scarcity of reports on prognostic factors for patients with RCC treated with ICIs. Therefore, the present study aimed to investigate the prognostic factors for patients with unresectable or metastatic RCC treated with ICIs.

## Patients and methods

### Patients

Forty-three patients treated with ICIs for unresectable or metastatic RCC were enrolled at the Kanagawa Cancer Center between January 2018 and October 2021. The patients were treated with ICIs (nivolumab, pembrolizumab, or avelumab) with or without other anticancer agents, including anti-cytotoxic T-lymphocyte antigen 4 (CTLA4) antibody, ipilimumab, or the tyrosine kinase inhibitor (TKI), axitinib. Peripheral blood (heparin-anticoagulated) was collected from these patients at the start of ICI treatment to measure soluble immune mediators. This study was approved by the Ethics Committee of the Kanagawa Cancer Center (#28–85), and written informed consent was obtained from all patients.

## Analysis of soluble immune mediators in the plasma

The plasma levels of soluble immune mediators before ICI administration were evaluated using a bead-based multiplex assay. In this assay, soluble immune mediators, including cytokines, chemokines, and growth factors, were measured in 50 μL aliquots of fourfold diluted plasma using the Bio-Plex 200 system (Bio-Rad Laboratories, Hercules, CA, USA). The Analyte Kit from Bio-Rad Laboratories was used to measure the following 73 soluble immune mediators; interleukin (IL)-1β, IL-2, IL-4, IL-6, IL-8, IL-10, IL-11, IL-12 (p40), IL-12 (p70), IL-16, IL-19, IL-20, IL-22, IL-26, IL-27, IL-28A, IL-29, IL-32, IL-34, IL-35, interferon (IFN)-α2, IFN-β, IFN-γ, tumor necrosis factor (TNF)-α, granulocyte macrophage colony-stimulating factor (GM-CSF), C–C motif chemokine ligand (CCL)1, CCL2, CCL3, CCL7, CCL8, CCL11, CCL13, CCL15, CCL17, CCL19, CCL20, CCL21, CCL22, CCL23, CCL24, CCL25, CCL26, CCL27, C-X-C motif chemokine ligand (CXCL)1, CXCL2, CXCL5, CXCL6, CXCL9, CXCL10, CXCL11, CXCL12, CXCL13, CXCL16, C-X3-C motif chemokine ligand (CX3CL)1, macrophage migration inhibitory factor (MIF), sCD30, sCD163, chitinase 3-like-1, gp130, IL-6Rα, soluble tumor necrosis factor receptor (sTNFR)1, sTNF-R2, a proliferation-inducing ligand (APRIL), B cell activation factor (BAFF), LIGHT, pentraxin-3, thymic stromal lymphopoietin (TSLP), TWEAK, osteocalcin, osteopontin, matrix metalloproteinase (MMP)1, MMP2, and MMP3.

## Statistical analysis

Progression-free survival (PFS) was calculated from the start date of ICI therapy to the date of progression, death, or last follow-up. Overall survival (OS) was calculated from the start date of ICI therapy to the date of death or last follow-up. Patients who were alive were censored on the date of the last contact. Immune-related adverse events (irAE) that were Grade 3 or higher or resulted in withdrawal or discontinuation were counted. The patients were dichotomized into high and low groups by setting the median value of each factor as the cut-off. PFS and OS were evaluated using the Kaplan–Meier method (log-rank test) and the COX proportional hazards model, with p < 0.05 set as the significance level. Statistical analyses were performed using SPSS ver. 24.0.

## Results

### Patient characteristics

**Fig. 1:**
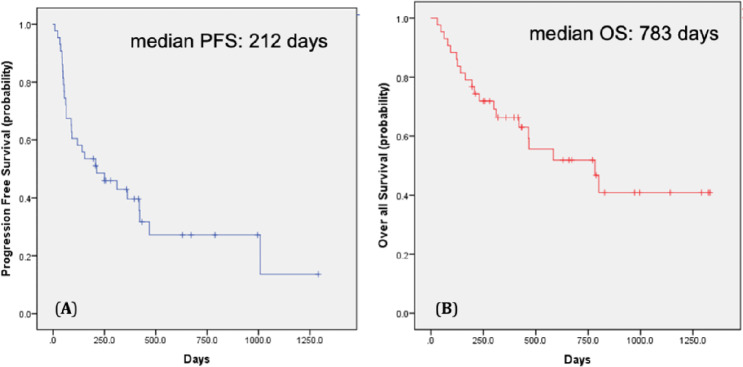
Kaplan–Meier estimates of cumulative PFS (**A**) and OS (**B**) in all patients with RCC

Patient characteristics are shown in Table 1. The median age was 66 years (32–86 years). Twenty patients were treated with ICIs as the 1st line, and 23 as the 2nd or subsequent lines. Fifteen patients were treated with nivolumab and ipilimumab, 23 with nivolumab alone, 4 with pembrolizumab and axitinib, and 1 with avelumab and axitinib. According to the IMDC classification, 7 patients were classified as poor risk, 30 as intermediate risk, and 6 as favorable risk. Among the patients, 26 experienced Grade 3 or higher irAEs or those related to ICIs, leading to treatment withdrawal or discontinuation. The most common cause was adrenal insufficiency, observed in 7 cases. The median observation period was 358 days (95% confidence interval [CI] 31–1332 days), the median PFS was 212 days (95% CI 14.2–409.7 days), and the median OS was 783 days (95% CI 374.5–1191.5 days). The Kaplan–Meier estimates of PFS and OS in all patients are shown in Fig. 1.

**Table 1:** Patient characteristics

Variables	Overall (*n* = 43)
Age (year), median (IQR)	66 (32–86)
*Sex*	
Male	35
Female	8
*Pathological tissue*	
Clear cell	39
Papillary type 2 + sarcomatoid change	1
Fumarate hydratase-deficient	1
Collecting duct	1
unclassified	1
*Treatment details*	
1st line	20
2nd line or more	23
*Medicated drugs*	
Nivolumab + ipilimumab	15
Nivolumab alone	23
Pembrolizumab + axitinib	4
Avelumab + axitinib	1
*IMDC classification*	
Poor	7
Intermediate	30
Favorable	6

## Significant prognostic factors in patients with unresectable or metastatic RCC treated with ICIs

**Fig. 2:**
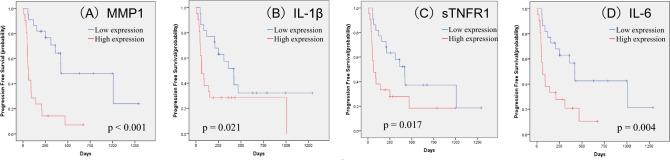
Kaplan–Meier estimates of cumulative PFS in the groups stratified by plasma levels of MMP1 (**A**), IL-1β (**B**), sTNFR-1 (**C**), and IL-6 (**D**). The cut-off values between the high and low groups were the median values. *p* values (log-rank test) are shown

**Fig. 3:**
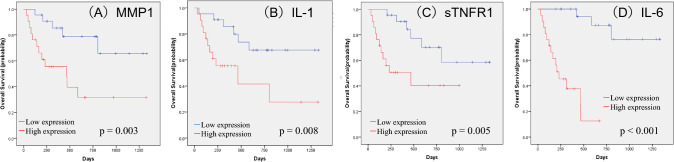
Kaplan–Meier estimates of cumulative OS in the groups stratified by plasma levels of MMP1 (**A**), IL-1β (**B**), sTNFR-1 (**C**), and IL-6 (**D**). The cut-off values between the high and low groups were the median values. *p* values (log-rank test) are shown

**Fig. 4:**
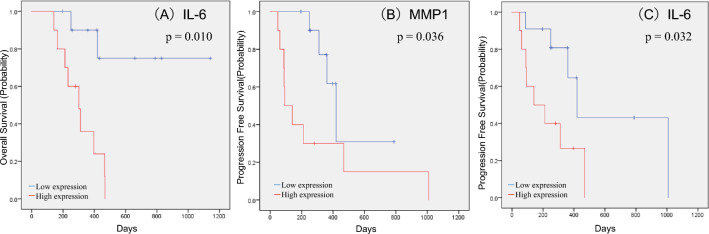
Kaplan–Meier estimates of cumulative PFS (**A**) and OS (**B**, **C**) in the group treated with 1st line ICIs (*n* = 20). In this group, patients were stratified by plasma levels of MMP1 and IL-6. The cut-off values between the high and low groups were the median values. *p* values (log-rank test) are shown

Prognostic significance in each soluble factor was evaluated using the Kaplan–Meier method with the log-rank test. We identified 12 factors, including MMP1 (p < 0.001), IL-1β (p = 0.021), sTNFR-1 (p = 0.017), IL-6 (p = 0.004), IL-10 (p = 0.001), IL-11 (p = 0.001), MMP2 (p = 0.034), TSLP (p = 0.037), CXCL13(p = 0.023), CCL11 (p = 0.007), CCL26 (p = 0.047), and CXCL2 (p = 0.014), that are significantly associated with PFS. Moreover, 7 factors, including MMP1 (p = 0.003), IL-1β (p = 0.008), sTNFR-1 (p = 0.005), IL-6 (p < 0.001), IFN-γ (p = 0.032), IL-35 (p = 0.027), and osteocalcin (p = 0.022), were significantly associated with OS. Significant differences in both PFS and OS were observed for MMP1, IL-1β, sTNFR-1, and IL-6. The Kaplan–Meier estimates of PFS and OS according to MMP1, IL-1β, sTNFR-1, and IL-6 levels are shown in Figs. 2 and 3. Multivariate analysis of these factors demonstrated that MMP1 was significantly associated with PFS (hazard ratio [HR] 5.305, 95% CI, 1.648–17.082; p = 0.005), whereas IL-6 was significantly associated with OS (HR 23.876, 95% CI, 3.426–166.386; p = 0.001) (Table 2). An additional analysis focusing on 20 cases within the 1st line of treatment exclusively was conducted. The median PFS was 361 days (95% CI 205–516 days), and the median OS was 466 days (95% CI 353.7–578.3 days). Notably, IL-6 was a significant factor in OS, and IL-6 and MMP1 were significant factors in PFS (Fig. 4).

**Table 2:** Multivariate analysis for PFS and OS

Variables		Multivariate (PFS)		Multivariate (OS)	
		HR (95% CI)	*p* value	HR (95% CI)	*p* value
MMP1 level, pg/mL	≧804.33 vs. < 804.33	5.305 (1.648—17.082)	0.005	0.562 (0.136—2.321)	0.426
sTNFR-1 level, pg/mL	≧5223.67 vs. < 5223.67	1.505 (0.646—3.507)	0.344	2.368 (0.735—7.631)	0.149
IL-1β level, pg/mL	≧6.23 vs. < 6.23	2.121 (0.934—4.819)	0.072	1.993 (0.597—6.651)	0.262
IL-6 level, pg/mL	≧21.47 vs. < 21.47	0.751 (0.24—2.347)	0.622	23.876 (3.426—166.386)	0.001

*HR* hazard ratio, *CI* confidence interval, *PFS* progression-free survival, *OS* overall survival, *ICI* immune checkpoint inhibitor, *MMP1* matrix metalloproteinase 1, *IL-6* interleukin 6

## Subgroup analysis

**Fig. 5:**
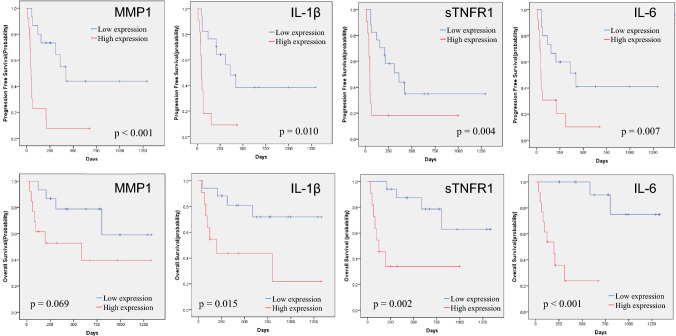
Kaplan–Meier estimates of cumulative PFS (upper raw) and OS (lower raw) in the subgroups treated with ICIs without ipilimumab (*n* = 28). In each subgroup, patients were stratified by plasma levels of MMP1, IL-1β, sTNFR-1, and IL-6. The cut-off values between the high and low groups were the median values. *p* values (log-rank test) are shown

**Fig. 6:**
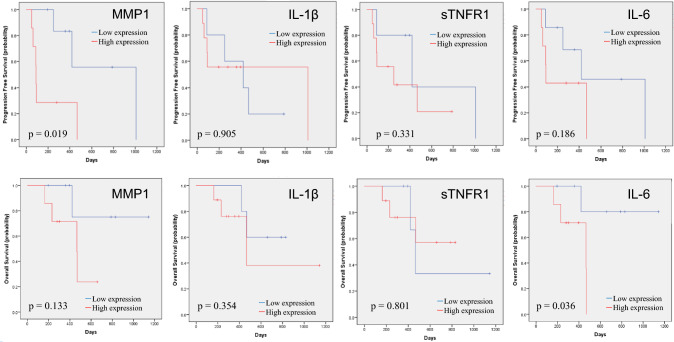
Kaplan–Meier estimates of cumulative PFS (upper raw) and OS (lower raw) in the subgroups treated with nivolumab and ipilimumab (*n* = 15). In each subgroup, patients were stratified by plasma levels of MMP1, IL-1β, sTNFR-1, and IL-6. The cut-off values between the high and low groups were the median values. *p* values (log-rank test) are shown

PFS and OS were analyzed in the subgroups treated with ICIs with (n = 15) or without (n = 28) ipilimumab. As shown in Fig. 5, in patients treated with ICIs without ipilimumab, IL-1β (PFS, p = 0.010; OS, p = 0.015), sTNFR-1 (PFS, p = 0.004; OS, p = 0.002), and IL-6 (PFS, p = 0.007; OS, p < 0.001) were significantly associated with both PFS and OS, whereas MMP1 was significantly associated with PFS (p < 0.001), but not with OS (p = 0.069). In contrast, in patients treated with both nivolumab and ipilimumab, significant differences were observed in MMP1 (p = 0.019) for PFS and IL-6 (p = 0.036) for OS, but not in other factors (Fig. 6).

## Discussion

In this study, among the 73 investigated plasma humoral factors, MMP1, IL-1β, sTNFR-1, and IL-6 were prognostic factors for both PFS and OS; higher expression of these factors was associated with worse PFS and OS. In addition, our multivariate analysis identified MMP1 and IL-6 as significant prognostic factors for PFS and OS, respectively.

MMP1 is an endopeptidase expressed in various cells such as fibroblasts, keratinocytes, endothelial cells, monocytes, and macrophages. The epithelial expression of MMP1 has been reported to inhibit mitochondrial function, increase HIF-1α expression, decrease the generation of reactive oxygen species, and contribute to proliferative, migratory, and antiapoptotic cell phenotypes [7]. MMP1 also mediates the invasion of circulating tumor cells into the tumor environment [8]. For example, MMP1 overexpression is associated with tumor invasion and metastasis and is involved in the pathogenesis of lung cancer [9]. In colorectal cancer, MMP1 promotes cancer cell growth by stimulating the cell cycle [10]. Notably, the expression of MMP1 is inversely associated with the infiltration of T cells and macrophages in cervical squamous cell carcinoma [11].

IL-6 is a multifaceted cytokine involved in various immune responses such as autoimmunity and antitumor immunity [12]. In the tumor immune microenvironment of RCC, IL-6 induces the expression of SOCS3 (suppressor of cytokine signaling-3), a negative regulator of cytokine signaling that promotes tumor cell invasion and metastasis. The inhibition of this cascade may prevent tumor cells from undergoing invasive metastasis and prolong prognosis [13]. Notably, IL-6 has also been reported as a prognostic predictor of ICI therapy in lung cancer and melanoma [14, 15]. In addition, combination therapy with ICIs and IL-6 inhibitors exhibits a decoupling effect on the antitumor effects and toxicity [16]. Moreover, CRP is a surrogate marker for IL-6 and has been reported to be a prognostic factor in previous reports, we also analyzed it in the present study; however, we observed no significant differences [6]. Given that IL-6 exhibited a considerably high HR for OS in our multivariate analysis, combination therapy with ICIs and anti-IL-6 antibodies may also prolong OS in patients with RCC.

IL-1β induces tumor angiogenesis through activation of the vascular endothelial growth factor pathway, increases immunosuppressive cells, and accelerates tumor invasion via secretion of GM-CSF and IL-6 [15] [17, 18]. IL-1β is also a prognostic factor in various cancers, including lung, breast, colon, gastric, and esophageal cancers [19] [20–24]. In RCC, IL-1β promotes the infiltration of myeloid-derived suppressor cells and tumor-associated macrophages and confers resistance to acquired and innate immunity. Thus, based on the results of this study, combination therapy with IL-1β inhibitors and ICIs may enhance antitumor effects in RCC [25].

In addition, sTNFR-1 inhibits the action of TNF-α by competitively preventing the binding of circulating TNF-α to the transmembrane form of TNFR-1. Because TNFα is a pleiotropic cytokine with several immunological roles [26], the inhibition of TNFα mediated by sTNFR-1 may suppress the antitumor activity of ICIs in patients with RCC.

High expression of MMP1 is a predictor of irAEs; high MMP1 expression is highly associated with irAEs in lung cancer [27]. The comprehensive exploration of the relationship between MMP1 and irAE is challenging. However, it is plausible that the involvement of MMP1 with monocytes may contribute to irAEs. High MMP1 expression may also indicate an elevated expression state of TNFα, GM-CSF, and IFN-γ [28, 29]. This collective upregulation of all these factors may be responsible for the complex activation of the immune response in vivo and the subsequent irAE induction following ICI administration, warranting future comprehensive investigations.

Notably, in the present study, the subgroup analysis demonstrated that the prognostic roles of each soluble factor may be different between the patients treated with ICIs with and without ipilimumab. In patients treated with both nivolumab and ipilimumab, IL-1β and sTNFR-1 were not significant prognostic factors for PFS or OS, suggesting that ipilimumab may be involved in tumor immunity by regulating the activity of IL-1β and sTNFR-1. The relationship between CTLA4 and IL-1β/sTNFR-1 remains unclear. However, IL-1β secretion is regulated by CTLA4-Ig fusion protein [30], and the administration of IL-1β inhibitors in a mouse model of hepatitis upregulates the expression of PD-1 and CTLA4 in the liver [31]. Moreover, TNFR-1 contributes to the termination of immune responses through its ability to induce apoptosis, and the possible involvement of CTLA4 in these apoptotic pathways has also been reported [32]. Given that CTLA4 inhibition may lead to apoptosis more readily in groups overexpressing IL-1β and sTNFR-1, combined treatment with nivolumab and ipilimumab may be recommended in patients with high IL-1β and sTNFR-1 expression levels in the plasma. However, these recommendations are speculative and require further experimentation and case series.

This study has several limitations. First, it was a single-center retrospective study with a limited number of patients and a short observation period. Second, the treatment lines and regimens used varied. Finally, patients with tumor types other than clear cell carcinoma were included in this study. Further studies with a larger number of patients on the same treatment lines and regimens are needed to investigate the prognostic roles of each factor more accurately.

In summary, this study demonstrated that MMP1, IL-1β, sTNFR-1, and IL-6 are prognostic factors in patients with RCC treated with ICIs. In particular, IL-6 was detected as an independent predictor of OS, and MMP1 was identified as an independent predictor of PFS in the multivariate analysis.

## Data Availability

The datasets used and analyzed in the current study are available from the corresponding author upon reasonable request.
